# Differential Cytokine Gene Expression According to Outcome in a Hamster Model of Leptospirosis

**DOI:** 10.1371/journal.pntd.0000582

**Published:** 2010-01-12

**Authors:** Frédérique Vernel-Pauillac, Cyrille Goarant

**Affiliations:** Institut Pasteur de Nouvelle-Calédonie, Nouméa, New Caledonia; University of Washington, United States of America

## Abstract

**Background:**

Parameters predicting the evolution of leptospirosis would be useful for clinicians, as well as to better understand severe leptospirosis, but are scarce and rarely validated. Because severe leptospirosis includes septic shock, similarities with predictors evidenced for sepsis and septic shock were studied in a hamster model.

**Methodology/Principal Findings:**

Using an LD50 model of leptospirosis in hamsters, we first determined that 3 days post-infection was a time-point that allowed studying the regulation of immune gene expression and represented the onset of the clinical signs of the disease. In the absence of tools to assess serum concentrations of immune effectors in hamsters, we determined mRNA levels of various immune genes, especially cytokines, together with leptospiraemia at this particular time-point. We found differential expression of both pro- and anti-inflammatory mediators, with significantly higher expression levels of tumor necrosis factor α, interleukin 1α, cyclo-oxygenase 2 and interleukin 10 genes in nonsurvivors compared to survivors. Higher leptospiraemia was also observed in nonsurvivors. Lastly, we demonstrated the relevance of these results by comparing their respective expression levels using a LD100 model or an isogenic high-passage nonvirulent variant.

**Conclusions/Significance:**

Up-regulated gene expression of both pro- and anti-inflammatory immune effectors in hamsters with fatal outcome in an LD50 model of leptospirosis, together with a higher *Leptospira* burden, suggest that these gene expression levels could be predictors of adverse outcome in leptospirosis.

## Author Summary

Leptospirosis is a widespread bacterial infection that is transmitted by soil or water contaminated by the urine of infected animals, or directly from these animals. It has highly diverse clinical presentations, making its differential diagnosis difficult. Though most cases are minor and self-resolving, there are also severe forms that include a sepsis pattern and multiple organ failure, and have possible fatal outcomes. Predictors of disease evolution and outcome are scarce, yet they would be very valuable to clinicians as well as to better decipher disease pathogenesis. In this study, we used a hamster model of leptospirosis to evaluate if immune genes were differentially expressed between individuals and if their expression levels could help forecast the outcome of the disease. We found that hamsters that later died from leptospirosis had significantly higher expression levels of both pro- and anti-inflammatory mediators compared to survivors. These results suggest that expression levels of these immune effectors might be helpful predictors of outcome in leptospirosis and that septic shock contributes to fatal leptospirosis.

## Introduction

Leptospirosis is the most widespread zoonosis occurring worldwide with possible fatal outcomes [1]. Though most often an endemic disease, epidemics have been associated with particular meteorological events [2]–[4] or clusters of cases related to occupations or leisure activities [5]–[8]. It is notably highly prevalent in tropical areas, but some of the clusters of cases have been reported in temperate countries [7],[8]. Its clinical presentation is highly variable and is often initially suggestive of influenza, malaria or dengue fever, making the differential diagnosis more hazardous in tropical countries, during dengue or influenza epidemics, or in areas of high malaria incidence [9]. However, because of a relatively high fatality rate in leptospirosis, increased medical care must be provided to some of the patients suffering leptospirosis. Validated prognostic factors to help forecast the evolution of a leptospirosis are scarce. Yet, they would be valuable for clinicians to decide whether their patients should only be treated with antibiotics, kept at the hospital in a standard unit or directed to an intensive care unit. Few data have been published addressing this question; furthermore, contrasting observations were obtained [10],[11].

Severe leptospirosis manifestations include acute renal failure, caused by acute interstitial nephritis and pulmonary haemorrhage. Spirochete invasion and toxicity of outer membrane components cause robust inflammatory host responses [12] leading to clinical manifestations reflecting a sepsis syndrome. This latter condition has been characterized as a dysregulation of the inflammatory response, including a massive release of pro-inflammatory cytokines that induces multiple organ dysfunctions. Concomitantly, compensatory mechanisms, mostly regulatory cytokine-mediated (although having protective effects to prevent overwhelming inflammation) may become deleterious and have been associated with an immune paralysis and poor outcome [13],[14].

Cytokines are potent, pleiotropic, non-antigen-binding polypeptides secreted by cells of the immune system and are responsible for cell activation, differentiation and proliferation after they act on their target cells via specific receptors primarily through autocrine and paracrine stimulation. Their expression levels are largely studied in the context of septic shock and severe sepsis both for their possible prognosis value [13], [15]–[17], as well as for a better understanding of sepsis physio-pathology [18]–[20]. Because of high similarities in clinical presentation of septic shock and severe leptospirosis, we hypothesized that strong similarities in immune gene expression between severe sepsis and severe leptospirosis could help predict the evolution of leptospirosis towards multiple organ failure or recovery.

Tumor Necrosis Factor α (TNFα) is a cytokine involved in early systemic inflammation that stimulates the acute phase reaction, in synergy with interleukin -1 (IL-1) and interleukin-6 (IL-6) [21]. Elevated plasma concentrations of TNFα have been associated with poor prognosis in sepsis, but also in patients with leptospirosis [10]. IL-6, one of the most important pro-inflammatory mediators of the acute phase response to pathogens, has been suggested to be a downstream mediator of TNFα and IL-1. However, it also regulates anti-inflammatory effectors by controlling the level of pro-inflammatory cytokines. Many studies were conducted to evaluate the value of circulating IL-6 concentrations as indicators of clinical outcome in patients with severe sepsis, correlating high levels with fatal outcomes. Interferon-γ (IFN-γ) is a pluripotent pro-inflammatory cytokine [22]. Its production was shown as dependent on IL-12p40 in human blood stimulated by *L. interrogans* [23] notably inhibiting Th2 cell activity. Cox-2, one of the two forms of cyclooxygenase (COX), is highly induced and rapidly produced in macrophages and endothelial cells in response to proinflammatory cytokines and may be responsible for the oedema and vasodilatation associated with inflammation. It is recognized that inflammatory mediators such as COX-2 but also nitric oxide, a derived product of inducible nitric-oxide synthase (iNOS), are responsible for the symptoms of many inflammatory diseases [24],[25]. Increased level of nitric oxide have notably been evidenced in the sera of patients with severe leptospirosis [26].

Anti-inflammatory effectors play an important role to counter-regulate the effects of pro-inflammatory cytokines. Interleukin-10 (IL-10) is classically described as an anti-inflammatory cytokine with pleiotropic effects in immunoregulation and inflammation by down-regulating the expression of Th1 cytokines [27]. An early imbalance of IL-10 in sepsis was shown to be associated with death despite TNFα production. [17]. Transforming Growth Factor β (TGF-β) is believed to be important in the regulation of the immune system by regulatory T cells; it notably acts by blocking the activation of lymphocyte- and monocyte-derived phagocytes and by controlling iNOS expression [28]. Together with IL-10, TGFβ is considered as contributing to the immunosuppression observed in septic shock [13],[14].

The hamster is considered as the most valuable animal model for human leptospirosis [29],[30]. This animal model is notably used to maintain virulence of *Leptospira* strains or isolates. It was also used in studies aiming at better deciphering the virulence and pathogenesis mechanisms or the host immune response to leptospirosis or to vaccine candidates [30]–[37]. Using this animal model, our group [38] notably demonstrated *in vivo* the expression of Th1 cytokines during acute leptospirosis.

The aim of our study was, using a LD50 model of leptospirosis in hamsters, to evaluate gene expression levels in individual animals. Additionally, the *Leptospira* burden in blood was also assessed because it was shown to have a prognostic value [39]. The immune gene expression levels and *Leptospira* burdens were compared according to the spontaneous outcome of the leptospirosis. Differential expression levels were observed that related to the outcome of the infection.

## Methods

### *Leptospira* strain and cultivation

The virulent *Leptospira interrogans* serovar Icterohaemorrhagiae strain Verdun, was obtained from the Reference Collection of the Institut Pasteur in Paris, France. Virulence was maintained by passages in Syrian golden hamsters (*Mesocricetus auratus*) and was regularly tested by lethal injection of 2×10^8^ leptospires intraperitoneally. An avirulent variant corresponding to an isogenic clone of this strain was derived from the virulent strain by *in vitro* high-passage.

Leptospires were cultivated in liquid EMJH (Ellinghausen McCullough Johnson and Harris) medium at 30°C under aerobic conditions [40]. The bacterial cell density of the cultures was assessed in a Petroff-Hausser counting chamber.

### Experimental infections

Specific pathogen-free animals which parents were initially purchased from Charles River Laboratories (Charles River Wiga GmbH, Sulzfeld, Germany) were bred at the Institut Pasteur of New Caledonia. All *in vivo* studies were carried out using five- to six-week-old outbred golden hamsters handled in individual cages. During preliminary experiments, we infected hamsters by intraperitoneal injection of various doses of live virulent *Leptospira* ranging from 2×10^7^ to 2×10^8^ per hamster. Hamsters were checked four times a day to evaluate the appearance of clinical signs, deep unconsciousness or recovery. Deeply unconscious animals that did not react to a tactile stimulus were considered dead and euthanized. Additional preliminary experiments included the determination of the time course of gene expression by sampling three individual infected hamsters at 0, 4, 8, 14 hrs then day 1 (D1), D2, D3, and D4 post-infection to determine the most relevant time point allowing to evaluate the expression level of as many relevant genes as possible for future experiments. Each LD_50_ experimental set was composed of six- to eighteen animals intraperitoneally infected with 10^8^ leptospires of a virulent culture in EMJH medium, and three or four negative controls injected with sterile EMJH medium. The study was made up of three independent experiments. Whole blood (400 µl) was collected on PAXgene blood RNA tubes (PreAnalytiX, Qiagen, Australia) by cardiac puncture under non lethal gas anaesthesia [29] on D3 after infection. Clinical symptoms and/or death were monitored four times daily for 21 days. Surviving animals at D21 were considered as spontaneously recovering. Negative controls and surviving animals were euthanized at D21. In order to evaluate the effect of the infective dose on the gene expression patterns, we used two other experimental infection models. We first injected five hamsters with a LD100 of the same virulent *Leptospira*, with 2×10^8^ leptospires injected per hamster using the same intraperitoneal route. Secondly, we injected hamsters with a similarly high dose (2×10^8^ leptospires per hamster) of the high-passage isogenic *Leptospira* variant, known not to induce any mortality. Control animals were similar to the LD50 experiments and were injected with an equal volume of sterile EMJH. Protocols for animal experiments were prepared and conducted according to the guidelines of the Animal Care and Use Committees of the Institut Pasteur and followed European Recommendation 2007/526/EC that provides “guidelines for the accommodation and care of animals used for experimental and other scientific purposes”. The protocol was validated before the start of the experiments by a scientific committee and an animal care committee of the Institute Pasteur in New Caledonia.

### Total RNA isolation and cDNA synthesis

Total RNA was isolated from whole blood not later than 24 hours post-collection using the PAXgene Blood RNA system (PreAnalytiX) according to manufacturer's instructions, then immediately frozen at −80°C until use. DNase-treated RNAs were used to synthesize cDNA with the Transcriptor First Strand cDNA Synthesis Kit using random hexamers as specified by the manufacturer (Roche Applied Science). To minimize variation in the reverse transcription reaction, all RNA samples from a single experimental setup were reverse transcribed simultaneously and in duplicate.

### Primers and standard curves

The sequences of all primers used in this study are listed in table 1. They were designed with the LightCycler Primer Probe Design Software 2.0 (Roche Applied Science), selected according to intron spanning and GC%, and synthesized by Proligo Singapore Pte Ltd (Biopolis way, Singapore).

**Table 1:** Primers used in this study, amplicon size and elongation time.

Primers	Accession number	Sequence	Amplicon size (bp)	Elongation time (sec)
TNF-α forward	AF046215	AACGGCATGTCTCTCAA	278	10
TNF-α reverse		AGTCGGTCACCTTTCT		
IFN-γ forward	AF034482	GACAACCAGGCCATCC	226	10
IFN-γ reverse		CAAAACAGCACCGACT		
TGF-β forward	AF046214	ACGGAGAAGAACTGCT	245	10
TGF-β reverse		ACGTAGTACACGATGGG		
IL-10 forward	AF046210	TGGACAACATACTACTCACTG	308	12
IL-10 reverse		GATGTCAAATTCATTCATGGC		
IL-1α forward	AB028235	AGTTCGTCCTGAATGATTCC	202	10
IL-1α reverse		TGGTCTTCACCCTGAGC		
IL-6 forward	AB028635	AGACAAAGCCAGAGTCATT	252	10
IL-6 reverse		TCGGTATGCTAAGGCACAG		
COX-2 forward	AF345331	CAACTCCCTTGGGTGTGA	173	8
COX-2 reverse		TCCTCGTTTCTGATCTGTCT		
β-actin forward	AF046210	TCTACAACGAGCTGCG	357	12
β-actin reverse		CAATTTCCCTCTCGGC		

External standard curves either for household or effector genes consisted of serial dilutions of specific purified DNA ranging from 10^7^ to 1 copies as described previously [38]. The copy number of each standard was calculated by standard methods using the Avogadro constant and the size of the amplified target as described [41]. Each standard curve was validated using established criteria (specific melting temperature, size of the PCR product, a mean error ≤0.03 and a slope near −3.3).

### Real-Time PCR amplification

PCR amplifications and analysis were achieved using a LightCycler 2.0 instrument (Roche Applied Science) with software version 4.05. All reactions were performed in duplicates with the LightCycler FastStart DNA Master SYBR Green I kit (Roche Applied Science) in a final 20 µl volume with 4 mM MgCl_2_, 0.5 µM of each primer and 2 µL cDNA or 2 µL DNA standard dilution. Cycle conditions were optimized for each target, either immune mediator or β-actin. Amplification conditions consisted of an initial pre-incubation at 95° for 10 min (polymerase activation) followed by amplification of the target cDNA for 45 cycles (95°C for 8 s, 60°C for 5 s and a variable extension time at 72°C). Extension periods varied for each PCR depending on the length of the expected amplicon (∼1 s/25 bp) as shown in table 1.

Leptospiraemia was also determined after cDNA amplification with a PCR specific of a 331 bp sequence of the *L. interrogans rrs* (16S) gene [42] using a LightCycler 480 II instrument with software version 1.5.0. Amplification reactions were performed in duplicates with the LightCycler 480 SYBR Green I Master kit in a final 10 µL volume with 0.5 µM of each primer, 1 µL cDNA as follow: a 10 min enzyme activation at 95°C then 50 amplification cycles, each made of 8 s at 95°C, 5 s at 62°C and 12 s at 72°C. A negative control with PCR-grade water instead of cDNA was included in each run. With either instrument, melting peaks were automatically plotted by the software and used to assess the specificity of the amplified product.

### Results expression and statistical analysis

Absolute quantification of each target was done using the comparative cycle threshold (*C*_T_) method: the concentration of a given target mRNA in any unknown sample was calculated by comparing its *C*_T_ with the corresponding standard curve. Relative expression was calculated as the ratio of the target mRNA copy number to β-actin mRNA copy number. This ratio was then normalized using the same ratio calculated in uninfected controls (used as calibrators). This expression of the results allows directly providing an n-fold change ratio in gene expression of the experimental animals compared to their control counterparts. In this study, β−actin was used as the household reference gene since former work demonstrated that no significant difference was observed using either HPRT or β−actin [38].

The outcomes were defined as the spontaneous outcome of the infection and were either death (“nonsurvivors”) or spontaneous recovery (“survivors”). The results of three independent LD50 experiments were pooled and compared according to the outcome using Student's t test and Kruskal-Wallis test on Stata SE/8.0 for Windows (Stata Corporation, Texas, USA). The overall survival curve for all LD50 experiments was also plotted with 95% confidence intervals using Stata SE/8.0.

## Results

### Preliminary and experimental infections results

**Figure 1:**
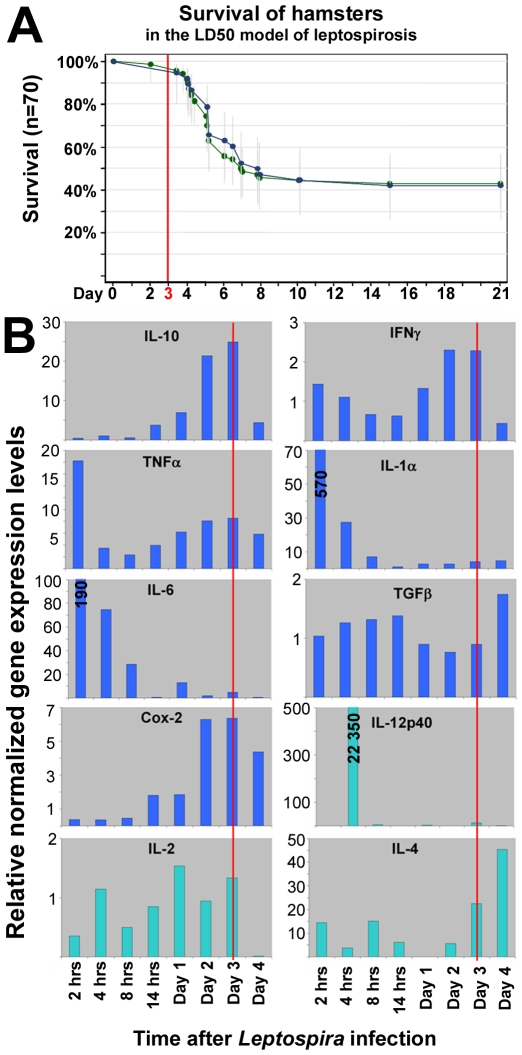
Preliminary experiments results leading to the selection of day 3 post-infection (as shown by the red lines) as an appropriate study time point. A. Time course of mortalities in the LD50 infection model (n = 70, green line) and in the animals sampled for the gene expression studies (n = 36, blue line). B. Relative normalized gene expression levels (see text) time courses (y-axes use different scales for different gene targets).

Preliminary experiments demonstrated that a dose of 10^8^ live virulent leptospires per hamster injected by the intra-peritoneal route led to ca. 50% mortality, a dose therefore used for our LD50 experiments. The first signs of illness (prostration and anorexia) were observed at day 3 post-infection. This dose was confirmed in further experiments as being a relevant and reproducible model of LD50 (figure 1A). The relative normalized gene expression levels at various time points are summarized in figure 1B. After a rapid and intense rise to its maximum, TNFα expression rapidly decreased before to slowly and regularly increase again up to D3. A similar pattern was observed for IL-1 and IL-6 with much a higher amplitude of regulation. After no significant modulation during the first 14 hrs post-infection, IL-10 and COX-2 were expressed at a maximum level around D3 after a steady increase notably for IL-10. IFNγ expression levels were poorly modulated along this time-course. However its maximum expression level became relatively stable around D3. Reproducible results were obtained for all these effectors with RNA extracted from 400 µL whole blood, except IL-2 and IL-4, because of their low mRNA copy numbers. Additionally, the peak of IL-12p40 gene expression was observed very early at 4 hours post-infection. Therefore these 3 latter effectors (IL-2, IL-4 and IL12p40) were not analysed in further studies.

Taken together, these results led to determine D3 as a relevant time point for future studies, a consensus when most of the relevant effectors could be efficiently monitored and the appearance of the first clinical signs before any mortality occurs.

In the LD100 experiment, all 5 infected hamsters died at D5. As expected, all hamsters infected with a similarly high dose (2×10^8^ per hamster) of the high-passage isogenic variant survived until D21 and were euthanized.

### Leptospira burdens and expression levels of some immune genes are different according to the outcome

**Figure 2:**
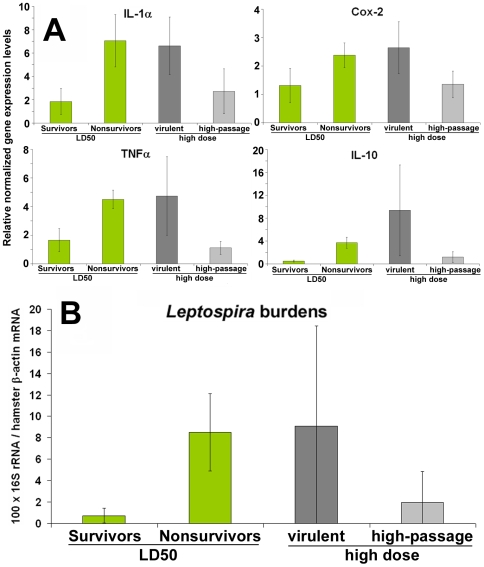
Mean expression levels of differentially expressed genes (relative normalized expression, see text). Expression levels are shown at day 3 post-infection in the LD50 *Leptospira* infection (according to the outcome) and after the injection of a high dose of a virulent or the high-passage variant (A) and *Leptospira* burdens (B) in infected hamsters. Error bars indicate 95% confidence intervals.

In total, 36 infected hamsters were included from our three independent LD50 infection challenges. Twenty two of them died at 6.1 (range 4.04–6.92) days post-infection (nonsurvivors), whereas 14 were considered as spontaneously recovering being alive at D21 (survivors). Gene expression levels evidenced that the pro-inflammatory cytokines IL-1α and TNFα but also the enzyme Cyclooxygenase-2 and the cytokine IL-10 were expressed at significantly higher levels (p<0.01, see table 2) in nonsurvivors when compared to survivors (Figure 2A).

**Table 2:** Relative normalized gene expression levels and *Leptospira* burdens of the hamsters at day 3 post-infections and p-values according to outcome in the LD50 experiments.

	Recovering LD50 (n = 14)	Dead LD50 (n = 22)	Student's t test	Kruskal Wallis	*Dead LD100 (virulent strain) (n = 8)*	*Avirulent strain (n = 10)*
	(10^8^ leptospires/hamster)		p (LD50 infections)		*(2×10^8^ leptospires/hamster)*	
TNFα	**1.65**	**4.51**	<0.001	<0.001	*4.74*	*1.11*
IL1a	**1.85**	**7.04**	0.002	<0.001	*6.62*	*1.20*
Cox2	**1.31**	**2.38**	0.007	0.005	*2.65*	*1.35*
IL10	**0.52**	**3.71**	<0.001	<0.001	*9.36*	*1.77*
TGFβ	1.07	1.06	0.975	0.338	*1.08*	*0.83*
IFNγ	0.97	1.45	0.086	0.067	*1.26*	*0.77*
IL6	5.48	4.36	0.666	0.135	*2.02*	*3.29*
Lepto 16S rRNA	**0.73**	**8.51**	0.002	0.0001	*9.07*	*1.97*

Considering the basic criterion that a 2-fold change in transcript abundance represents differential expression [43],[44], the gene expression levels in survivors were not significantly different from controls (i.e. relative normalized gene expression levels in the range 0.5–2, see table 2).

As expected, the live *Leptospira* burdens, as evaluated by the ratio of *Leptospira* 16S rRNA to hamster β-actin, were nil in controls and were also significantly (p<0.01, see table 2) lower in spontaneously recovering survivors (figure 2B).

### Some genes are not much modulated 3 days after *Leptospira* LD50 infection

**Figure 3:**
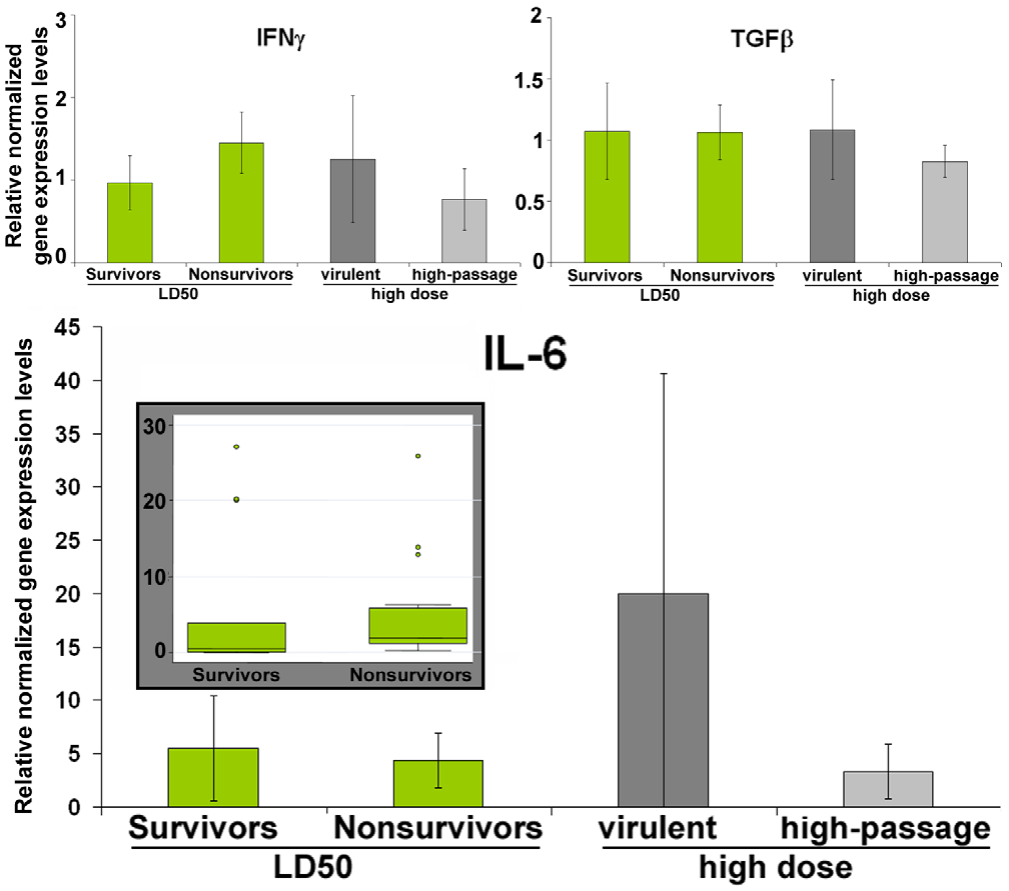
IFNγ, TGFβ and IL-6 gene expression levels at day 3 post *Leptospira* infection (relative normalized expression, see text). Error bars indicate 95% confidence intervals. The insert shows the high variability observed in IL-6 gene expression levels.

Contrastingly, the expression of the cytokines IFNγ and TGFβ appeared poorly modulated, their expression levels in infected animals being not significantly different from that in control animals (ratio not different from 1). Additionally, no difference in expression levels was observed between survivors and nonsurvivors after the *Leptospira* LD50 challenge (table 2 and figure 3). Though IL-6 is notably induced as a response to the LD50 infectious challenge (i.e. ratio significantly higher than 1.0), similar levels (p>0.1, see table 2) were observed in hamsters whatever the outcome of the LD50 infection. However and interestingly, two out of our three independent experiments, higher IL-6 expression levels were observed in nonsurvivors in two out of our 3 independent experiments and a very high expression level in a few survivors accounted for the similar average expression (see figure 3 insert).

### Comparisons with LD100 and a similarly high dose of an avirulent variant

Hamsters infected with a high (LD100) dose of virulent *Leptospira* displayed a gene expression pattern very similar to the one observed in nonsurvivors after the LD50 infection challenge. They displayed a similarly increased gene expression of IL-1α, TNFα and Cox-2 and a very similar *Leptospira* burden (figure 2). Their mean IL10 gene expression level was higher than in nonsurvivors after the LD50 challenge but this difference was not significant, due to high inter-individual variability. IFNγ and TGFβ expression levels were very poorly modulated, again not significantly different from uninfected controls (figure 3). Similar to IL-10, IL-6 gene expression was largely increased compared to animals infected with a lower dose but a high inter-individual variability was also noted.

In hamsters infected with a similarly high dose of the high-passage non-virulent *Leptospira* variant, the gene expression pattern was similar to the one displayed by survivors of the LD50 challenge. Surprisingly, a leptospiraemia was still observed at D3 in most of the animals, though no clinical sign was noted and no mortality occurred.

## Discussion

We first developed a LD50 model of leptospirosis in hamsters. Used together with a non-lethal blood sampling technique, it allowed the acquisition of individual gene expression patterns during the course of acute leptospirosis. Using this model, we demonstrated that the expression of some immune genes in blood, together with the *Leptospira* burden in blood of infected animals could be correlated with the outcome of the infection.

The hamster is recognized as a good animal model for severe human leptospirosis [29]. Using the virulent *Leptospira interrogans* Icterohaemorragiae strain Verdun [45],[46], we determined the dose of 10^8^ live leptospires injected via the intra-peritoneal route as leading to ca. 50% mortality. This challenge technique proved to be reproducible in 5 to 6-week old Syrian hamsters and was used for our study. When infected this way, the first clinical signs in hamsters held in individual cages (anorexia, then prostration and ruffled fur) were observed from 3 days post-infection on. This 3-day post-infection time point was also shown, in another experimental hamster model of leptospirosis, to be the time point for the first detection of *Leptospira* mRNA in target organs and a relevant time point for immune gene expression studies [47]. The use of a non-lethal sampling technique together with the follow up of individual hamsters allowed relating the gene expression levels observed with the individual outcome. We additionally conducted two experimental infection experiments for comparison purpose, one using a high dose of the virulent strain (2×10^8^ live leptospires via the intra-peritoneal route) leading to 100% mortality and a similarly high dose of an isogenic avirulent variant causing no mortality.

During these preliminary experiments, we determined that a 400 µL blood collection under gas anesthesia at this time point would not be too deleterious to the animals and was sufficient to allow the extraction of mRNA in adequate quantity and quality for gene expression studies. Based on our previous knowledge [38] and with additional preliminary experiments, we determined that TNFα, IL-1α, IL-6, IL-10, IFNγ, TGFβ and Cox2 gene expression levels could successfully be quantified using this experimental procedure. These targets were chosen for their relevance in studying our hypothesis of similarities between severe leptospirosis and septic shock. Only those evidencing a significant number of mRNA copy numbers at this time point, therefore allowing accurate determination of the gene expression levels, were studied.

The *Leptospira* burden was reported to be of prognostic value in human leptospirosis [39]. In our study, it was evaluated by q-RT-PCR targeting the 16S-rRNA allowing to evaluating the burden of mostly live leptospires, bacterial rRNAs being very short-lived when cells have reduced activity or die [48],[49]. Using a ratio of *Leptospira* 16S rRNA copy number to host β-actin mRNA copy number allowed a comparison between individuals. As expected and observed in humans [39], significantly higher *Leptospira* burdens were noticed in nonsurvivors. Interestingly, a leptospiraemia was still observed at D3 in animals infected with a high dose of the avirulent *Leptospira* variant, suggesting that this high-passage variant, though not lethal, has retained some degree of pathogenicity. This also suggests that leptospiraemia might be strain- and virulence-dependent, possibly jeopardizing its use as a tool for prognosis, when the virulence of the infecting strain is not known.

The immune response to an infection is nowadays considered as precisely modulated rather than simply induced. Cytokines expression levels are largely studied in the context of septic shock and severe sepsis both for their possible prognostic value [13], [15]–[17] and as a way to improve our understanding of host-pathogen interactions. Actually, sepsis is now recognized as associated with an exacerbated production of both pro- and anti-inflammatory cytokines and the prognostic value of some of these is widely recognized [20].

Using reverse transcription-real-time PCR, the transcripts can be quantified directly in a biological sample, providing information about the *in vivo* immune response mechanisms of the individual. Studying the immune response is only possible at the transcriptional level in our animal model, due to the lack of tools for assessing serum conentrations of immune efectors. however, it is also probably more sensitive to evaluate the fine-tuning of the immune response because the amount of circulating cytokines only represents a minor part of the total amount of cytokines produced [50].

Our results in the LD50 model evidenced differential gene expression according to the outcome. TNFα, IL-1α, Cox-2 were expressed at significantly higher levels in nonsurvivors than in spontaneously recovering hamsters. Using the other two infection models, the results demonstrated similar gene expression levels in animals challenged with a LD100 and in nonsurvivors after a LD50 challenge on one hand, and on the other hand in survivors after a LD50 and animals infected with a high dose of the avirulent variant, both actually surviving. These similarities using different doses and strains confirm the validity of our results.

Our IL-1 RT-PCR targets IL1α, one of the two main active forms in the IL-1 family. IL-1β is most often considered as the prototypic IL-1 effector because it is released in the bloodstream, whereas Il-1α mostly remains cytosolic with an autocrine activity or is bound to the cell surface. Though IL-1β was more frequently considered as an indicator of Il-1 activity, IL-1α was shown to have an action very similar to the action of the more largely studied IL-1β. Furthermore, it was shown that its gene expression is quite similarly regulated [51]–[53]. TNFα and IL-1 are prototypic pro-inflammatory mediators that have been reported to have a prognosis value in sepsis [16],[54], even if their clinical relevance was also questioned [55]. Interestingly, TNFα was also reported to have a similar prognosis value in leptospirosis [10], though these results were not confirmed in other studies [11]. Cox-2 is a highly inducible enzyme involved in the early phase of the inflammatory response. Notably induced by IL-1 and TNFα through the NFκB pathway, its induction can be considered as an end-result of the initial pro-inflammatory response. Interestingly, a significant induction of Cox-2 was observed only in nonsurvivors whatever the infective dose. This further suggests the probable contribution of a sepsis-like mechanism in severe leptospirosis. IL-10 is expressed at higher levels in nonsurvivors compared to the survivors in the LD50 model. These results are in agreement with several studies showing an exacerbated production of anti-inflammatory cytokines resulting in aggravation of a systemic disease and adverse outcome in febrile patients [14],[17]. The decreased production of Th1 cytokines in many cellular types was reported as an IL-10-induced adaptative immune response, by interfering on antigen-presenting cells and T cells, possibly via inhibition of NFκB nuclear translocation [14],[56]. This immunoregulatory role of IL-10 was clearly established after an anergy was observed in stimulated T cells in the presence of IL-10. Moreover, Il-10 production in innate immune response to a stimulus promotes the expansion of regulatory T cells, amplifying the anti-inflammatory effect of IL-10 [13],[14],[56].

However, the results observed with the other two infection models suggest a possible effect of the infective dose on IL-10 expression levels, higher levels being noted in animals infected with a high *Leptospira* dose (either virulent or not) when compared to their respective counterparts with the same outcome in the LD50 model. The IL-10/TNFα ratio has been proposed as a prognosis indicator in sepsis [17],[54] and in leptospirosis [57]. A high IL-10/TNFα ratio was reported as correlated with poor outcome in septic patients, contrary to the opposite results obtained in one limited study reported in leptospirosis [57]. This ratio was generally regarded as reflecting a persistent secretion of IL-10 at later time points, when concomitant down-regulation of TNFα occurs. From our experiments, this ratio, at least at the transcriptional level, does not appear relevant, both cytokines being induced in animals with fatal outcome, at least on the basis of our D3 time-point.

In our LD50 experiments, IL-6, often reported as the best marker of the severity of infectious (or even non-infectious) stress in humans and to have a prognosis value in sepsis [20], was not differentially expressed according to the outcome. However, our results merely reflect a very high inter-individual variability in IL-6 expression. Contrastingly, IL-6 expression in hamsters infected with (and dying from) a LD100 of *Leptospira* actually showed a highly increased expression, though again with a very high variability. Similarly, high fluctuations of bioactive IL-6 levels were reported in serum from septic patients [58]. This high variability would also be limiting the value of IL-6 as a predictor in leptospirosis.

In our model, IFNγ and TGFβ had no prognostic value and were similarly expressed in infected hamsters whatever the dose or the outcome. Interestingly, the gene expression of IFNγ and TGFβ appeared not being significantly regulated based on the common 2-fold variation criterion [43],[44]. Because IFN-γ gene expression is antigen-presenting cells dependent, we could hypothesize that high Il-10 levels limited its expression, though it could have been beneficial for an optimal defense against infection.

However, some cytokines have been shown not to be regulated at a transcriptional level and post-transcriptional regulation also plays a major role in cytokine cascades even after a transcriptional regulation has occurred. Unfortunately in the hamster, our animal model, no tool is available to evaluate the serum concentrations of the effectors studied. On one hand, gene expression techniques allow a rapid and highly sensitive study of immune gene transcriptional regulation, notably because the circulating part of cytokines is considered as being the “tip of the iceberg” [50]. On the other hand, some immune mediators of the septic shock are known to be poorly regulated at the transcriptional level and can therefore not be studied in our model. As an example, High-mobility group box (HMGB)-1 is primarily known as a nuclear DNA-binding protein with a transcription regulatory activity, but can also be excreted by stimulated macrophages, then displaying a cytokine activity, notably inducing the release of TNFα and IL-6 [59]. It has been proposed as a prototypic late mediator of inflammation in severe sepsis [60], its delayed and prolonged release in established sepsis rising an increasing interest as a prognostic indicator or a therapeutic target in late-phase inflammation processes. Its usefulness as a prognostic indicator or as a therapeutic target remains unexplored in leptospirosis.

Though obtained in an animal model with *Leptospira* strains of known and relatively low virulence, these encouraging results prompted us to initiate a clinical study aiming at investigating the prognostic value of these effectors in patients with confirmed leptospirosis. Cytokines will similarly be studied at the gene expression level, but also by measuring their serum concentration levels, a technique much easier transmissible to health centers. This study is currently underway.
